# The value of automated breast volume scanner combined with virtual touch tissue quantification in the differential diagnosis of benign and malignant breast lesions: A comparative study with mammography

**DOI:** 10.1097/MD.0000000000025568

**Published:** 2021-04-23

**Authors:** Junli Wang, Hongjie Fan, Yuting Zhu, Chunyun Shen, Banghong Qiang

**Affiliations:** a Department of Ultrasound, Wuhu No. 2 People's Hospital, Wuhu, Anhui 241001; b Department of Radiology, Sir Run Run Shaw Hospital, Zhejiang University School of Medicine, Hangzhou, Zhejiang 310016, China.

**Keywords:** automated breast volume scanner, breast lesions, mammography, virtual touch tissue quantification

## Abstract

This study aimed to evaluate the diagnostic value of automated breast volume scanner (ABVS) combined with virtual touch tissue quantification (VTQ) in the differential diagnosis of breast lesions.

In this retrospective study, 183 patients (mean age, 49.8 ± 8.2 years) with 218 breast lesions underwent ABVS, VTQ, and mammography (MG). All lesions were confirmed by postoperative histopathology. A logistic regression model was constructed to generate a receiver operating characteristic (ROC) curve, calculate the area under the ROC curve (AUC), and compare and evaluate the diagnostic performance of ABVS, VTQ, MG, and ABVS combined with VTQ (ABVS-VTQ).

The sensitivity, specificity, and accuracy of ABVS, VTQ, MG, and ABVS-VTQ in diagnosing breast lesions were 94.01% (110/117), 96.03% (97/101), and 94.95% (207/218); 80.34% (94/117), 94.05% (95/101), and 86.69% (189/218); 70.08% (82/117), 68.31% (69/101), and 69.26% (151/218); and 96.58% (113/117), 96.03% (97/101), and 96.33% (210/218), respectively. The AUC of ABVS-VTQ was higher than that of the other examinations alone. The detection rate of ABVS (100%, 218/218) was higher than that of MG (78.89%, 172/218), and the difference was statistically significant (*χ*^2^ = 51.426, *P* < .001).

The combined application of ABVS and VTQ can improve the accuracy and specificity of the diagnosis and is a promising ultrasound method for the differential diagnosis of breast lesions.

## 1 Introduction

In recent years, the incidence and prevalence of breast cancer have continued to rapidly increase, and the onset age tends to be younger.^[1]^ As a main cancer that can lead to death in women,^[2]^ breast cancer has become a serious threat to women's health. Therefore, the early detection and diagnosis of breast tumors are essential for improving the prognosis of patients with such diseases.

Mammography (MG) has served as a primary screening method for the diagnosis of breast cancer.^[3]^ MG has a high detection rate of microcalcification but is not sensitive in the diagnosis of dense breasts. Furthermore, as a radiological examination method, MG is not recommended for pregnant women or women aged <35 years.^[4]^ The sensitivity of MG can be as low as 48% in extremely dense breasts.^[5]^ One of the most widely used methods for diagnosing breast lesions in clinical practice is handheld ultrasound (HHUS), which can provide a preliminary diagnosis based on the morphological features of breast lesions. However, there is a considerable overlap between benign and malignant lesions, rendering a specific qualitative diagnosis difficult to achieve.^[6]^ Although HHUS is an affordable and practical method, it has the limitations of poor reproducibility and operator variability.^[7]^ The newly developed automated breast volume scanner (ABVS) can provide additional information regarding morphological features on the coronal plane, which can overcome the above shortcomings in the preoperative diagnosis of breast lesions.^[8]^ The retraction phenomenon on the coronal plane of ABVS is regarded as a reliable and prominent diagnostic feature in the differentiation of benign and malignant breast masses and has a high diagnostic accuracy in breast malignancy.^[9,10]^ ABVS has been proven as an adjunct for MG for screening, suggesting that the combination of ABVS and MG can significantly increase the detection rate of breast lesions compared with MG alone.^[11,12]^ The United States Food and Drug Administration (FDA) has approved its use in screening women with dense breast parenchyma.^[13]^

Acoustic radiation force impulse (ARFI) is a novel recently developed elastography imaging technique based on the assessment of elastic properties using acoustic pulse for the following 3 types of diagnosis: virtual touch tissue imaging (VTI), virtual touch tissue quantification (VTQ), and virtual touch tissue imaging quantification (VTIQ).^[14,15]^ The propagation velocity of waves is an intrinsic and reproducible property of tissue, and tissue quantification using the ARFI technology could generate objective and reproducible data. The stiffer the tissue through which the shear waves pass, the greater the shear velocity. The time to peak displacement at each lateral location is defined as the shear wave velocity (SWV, m/s), which is the quantitative form of VTQ.^[16]^ The SWV of soft tissue is slower than that of hard tissue, which is an objective indicator of tissue stiffness. According to previous reports, VTQ has been used for the diagnosis of breast lesions.^[17,18]^ To the best of our knowledge, both ABVS and VTQ are new ultrasonography imaging techniques, but these techniques have rarely been combined for the differential diagnosis of benign and malignant breast lesions. The current study was designed to investigate the value of ABVS combined with VTQ (ABVS-VTQ) and MG in the diagnosis and differential diagnosis of benign and malignant lesions.

## 2 Materials and methods

Ethical approval was provided by the institutional review board (IRB) of Wuhu No. 2 People's Hospital, and informed consent was obtained from all patients. This retrospective study was conducted in compliance with the Helsinki Declaration, and all individuals’ information was strictly kept confidential and anonymous in the manuscript.

### 2.1 Patients

From January 2018 to August 2019, 183 female patients with a total of 218 lesions (age range, 37–81 years; average, 49.8 ± 8.2 years; lesion diameter, 0.7–5.7 cm) underwent surgical resection due to suspicious breast lesions or upon request by patients in our hospital. All lesions were confirmed by postoperative histopathology. The inclusion criteria were as follows:

(a) age ≥ 35 years
(b) no previous lesion-related treatment.

**Figure 1:**
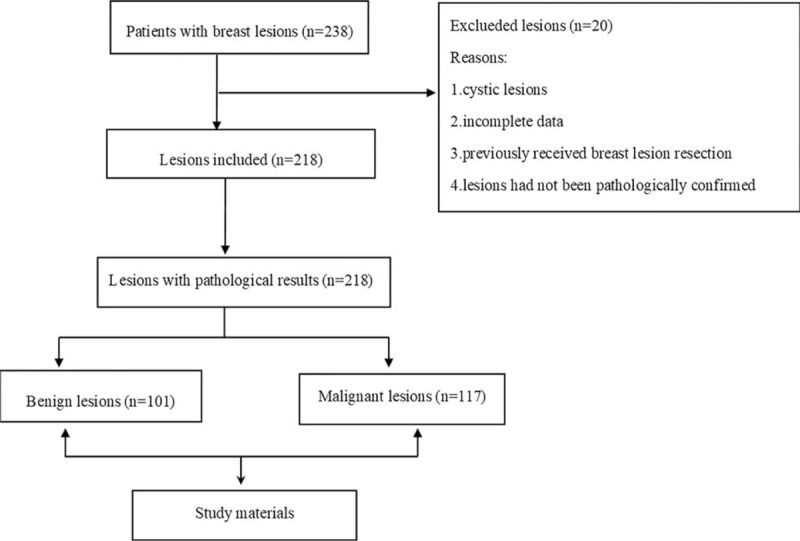
Flow chart and exclusion criteria of this study.

The following patients were excluded:

(a) patients with cystic lesions;
(b) patients with incomplete data;
(c) patients who previously underwent breast lesion resection; and
(d) patients whose lesions were not pathologically confirmed (Fig. 1).

### 2.2 Machine and operating methods

#### 2.2.1 ABVS examination method

ABVS was performed in all patients after HHUS and carried out using the ACUSON S2000 Automated Breast Volume Scanner (ABVS; Siemens Medical Solutions, Mountain View, CA), which includes a 15-cm-wide linear array transducer with a 5 to 14 MHz bandwidth, by a single technologist with at least 8 years of experience in operating the ABVS. All patients were in a supine or lateral position based on the location of the target lesions. The scanning orientation included anterior–posterior, lateral, and medial. In larger breasts, additional inferior and superior section scans were performed. Then, the gathered data were transferred to the ABVS workstation to obtain basic planar images, and three-dimensional reconstruction was performed based on the basic images of the whole breast, including those on the vertical and coronal planes. The analysis of the ABVS images was based on the characteristics of breast lesions, including shape, margin, echo pattern, orientation, posterior features, and microcalcification.

#### 2.2.2 VTQ examination method

VTQ was performed after selecting the optimal gray scale ultrasound image. During the examination process, the patient was asked to hold her breath for 3 to 5 seconds, and the transducer was not moved once placed on the breast with very slight pressure to ensure complete contact with the skin surface. The region of interest (ROI) was marked within the target lesion while avoiding calcified or cystic areas and necrotic tissue.

The SWV was measured automatically by the software, and the numeric values of the SWV (measured in meter/second) were calculated and posted on the monitor. The machine SWV range was set from 0 to 9 meter/second. When the measured value exceeded the tolerable range of the system for SWV calculation or when the tissue inside the ROI was heterogeneous or contained liquid components, the SWV may have been displayed as “X.XX.”^[19]^ When multiple measurements were shown as “X.XX”, all SWV values were recorded as “9 meter/second.”^[20]^ The SWV was measured 5 times per lesion (internal value), and the average of these 5 measurements was used as the SWV in this study.

#### 2.2.3 MG examination method

Mammography was performed using the Senographe DS system (GE Healthcare, Waukesha, WI) by a board-certified technician. Lateral, internal, and external oblique and axial images of both breasts were obtained from all patients.

#### 2.2.4 Image analysis and classification of lesions

The results of each method were interpreted and finally categorized into 6 categories according to the American College of Radiology (ACR) Breast Imaging Reporting and Data System (BI-RADS) as follows: 2 (benign); 3 (probably benign); 4A (low suspicion); 4B (intermediate suspicion); 4C (moderate suspicion); or 5 (highly suggestive of malignancy). In our study, benign lesions were considered BI-RADS categories 2 to 4A, and malignant lesions were considered categories 4B to 5, while BI-RADS categories 0 (incomplete), 1 (negative), and 6 (known biopsy-proven malignancy) were already excluded from this study. The image data from the ABVS were independently evaluated by 2 radiologists who were specialized in breast imaging with more than 6 years of experience. The acquired MG data were assigned at a separate workstation and evaluated by 2 radiologists with 10 years of experience in breast imaging.

### 2.3 Statistical analysis

SPSS 24.0 software (SPSS for Microsoft Windows, version 24.0; SPSS, Chicago, IL) was used for the statistical analysis. A binary logistic analysis was executed to perform a stepwise logistic regression analysis and generate a new variable PRE for each individual predicted probability. The new variable PRE was set as the test variable, and the diagnosis was set as the state variable for the receiver operating characteristic (ROC) curve analysis and calculation of the area under the ROC curve (AUC). The AUC values for each method were constructed and compared with the *Z* test, and the optimal cutoff value for the SWV was obtained. Count data are expressed as the number of cases or percentages. In all tests, *P* < .05 was considered statistically significant.

## 3 Results

### 3.1 Pathology results

In this study, 218 cases of pathologically confirmed breast lesions (101 cases of benign lesions and 117 cases of malignant lesions) from 183 patients were included. The pathological classification of these lesions is shown in Table 1.

**Table 1:** Pathologic result of breast lesions.

Benign lesions	n	Malignant lesions	n
Fibroadenoma	78	Invasive carcinoma of no special type	105
Intraductal papillomas	6	Carcinoma in situ	8
Benign phyllodes tumor	7	Mucinous carcinoma	1
Adenopathy with ductal epithelial hyperplasia	5	Medullary carcinoma	1
Adenopathy with distention of catheter	3	Intraductal papillary carcinoma	1
Granulomatous inflammation	1	Solid papilllary carcinoma	1
Fat necrosis	1		
Total	101		117

### 3.2 Comparison with the postoperative pathological diagnosis

The mean SWV values of the malignant lesions (7.60 ± 2.61 meter/second) were significantly higher than those of the benign lesions (2.69 ± 1.39 meter/second) (*t* = 13.893, *P* < .05; Table 2). The ROC curve was analyzed, and the optimal diagnostic cutoff value was obtained (Table 2). In this study, when the optimal diagnostic cutoff value was 3.96 meter/second, the sensitivity, specificity, and accuracy of VTQ in the diagnosis of the breast lesions were 80.34% (94/117), 94.05% (95/101), and 86.69% (189/218), respectively. The sensitivity, specificity, and accuracy of ABVS, ABVS-VTQ, and MG in the diagnosis of the breast lesions are shown in Table 3.

**Table 2:** Comparison of the mean SWV values of benign and malignant lesions.

	n	SWVmin
Benign lesions	101	2.69 ± 1.39 m/s
Malignant lesions	117	7.60 ± 2.61m/s
*t* value		13.893
*P* value		<.05

**Table 3:** Comparison of the “retraction phenomenon” on the coronal plane of ABVS and pathological results in breast lesions.

ABVS	Pathology		n
	Malignant lesions	Benign lesions	
+	74	4	78
−	43	97	140
	117	101	218

### 3.3 Comparison of the characteristics of benign and malignant breast lesions on coronal ABVS images

The incidence of the “retraction phenomenon” in the malignant breast lesions was higher than that in the benign breast lesions, and the difference was statistically significant (*χ*^2^ = 34.467, *P* < .001; Table 3).

### 3.4 Comparison of the detection rates of breast lesions between ABVS and MG

The detection rate of breast lesions using ABVS (100.00%) was higher than that of MG (78.89%), and the difference was statistically significant (*χ*^2^ = 51.426, *P* < .001; Table 4).

**Table 4:** Comparison of ABVS and MG for detection rates of breast lesions.

Inspection method	n	Detection rates (%)
ABVS	218	100.00 (218/218)
MG	172	78.89 (172/218)

### 3.5 Comparison of the detection rates of microcalcification in malignant breast lesions between ABVS and MG

The detection rates of microcalcification in the malignant breast lesions using ABVS and MG were 41.88% and 44.44%, respectively, but the difference was not statistically significant (*χ*^2^ = 0.157, *P* = .692; Table 5).

**Table 5:** Comparison of ABVS and MG for the detection rates of microcalcification in malignant breast lesions.

Inspection method	n	Detection rates (%)
ABVS	117	41.88 (49/117)
MG	117	44.44 (52/117)

### 3.6 ROC curve analyses of the diagnostic performance of different methods

**Figure 2:**
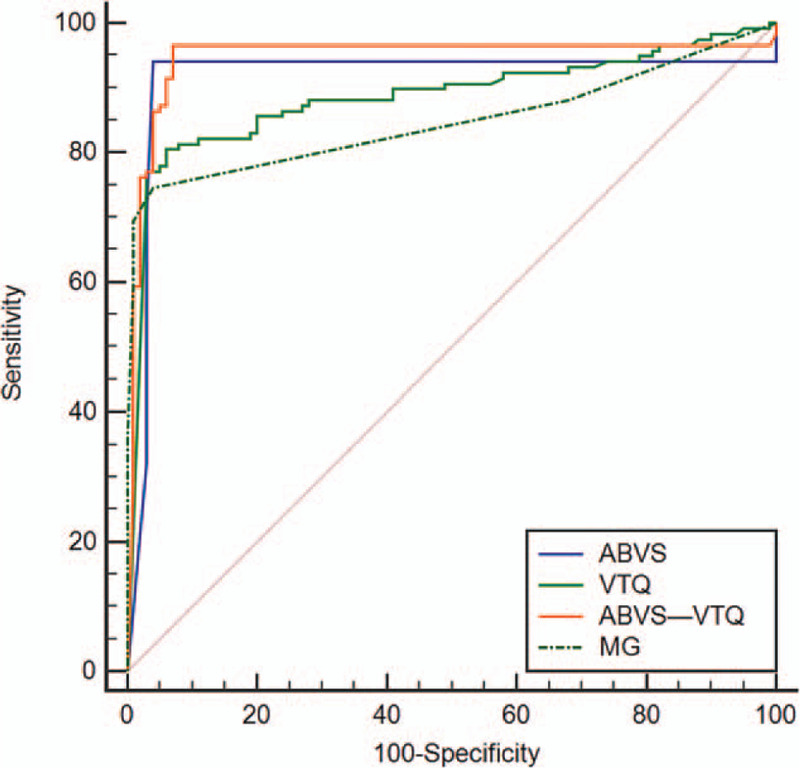
ROC curve of benign and malignant breast lesions diagnosed by each method.

ROC curves of the diagnostic performance of different methods, including ABVS, VTQ, MG, and ABVS-VTQ were constructed. The AUC of each inspection method was compared. There was no significant difference in the AUC among ABVS, VTQ, and MG (*P* > .05), while the AUC of ABVS-VTQ was larger than that of ABVS, VTQ, and MG (*P* < .05), indicating that the diagnostic value of ABVS-VTQ was higher than that of each examination method alone (Table 6 and Fig. 2).

**Table 6:** Diagnostic performance of each method (%/n).

Method	Sensitivity	Specificity	Accuracy	AUC	95%CI
ABVS	94.01 (110/117)	96.03 (97/101)	94.95 (207/218)	0.916	0.871, 0.949
VTQ	80.34 (94/117)	94.05 (95/101)	86.69 (189/218)	0.886	0.837, 0.925
ABVS+VTQ	96.58 (113/117)	96.03 (97/101)	96.33 (210/218)	0.948	0.909, 0.973
MG	70.08 (82/117)	68.31 (69/101)	69.26 (151/218)	0.848	0.793, 0.893

## 4 Discussion

**Figure 3:**
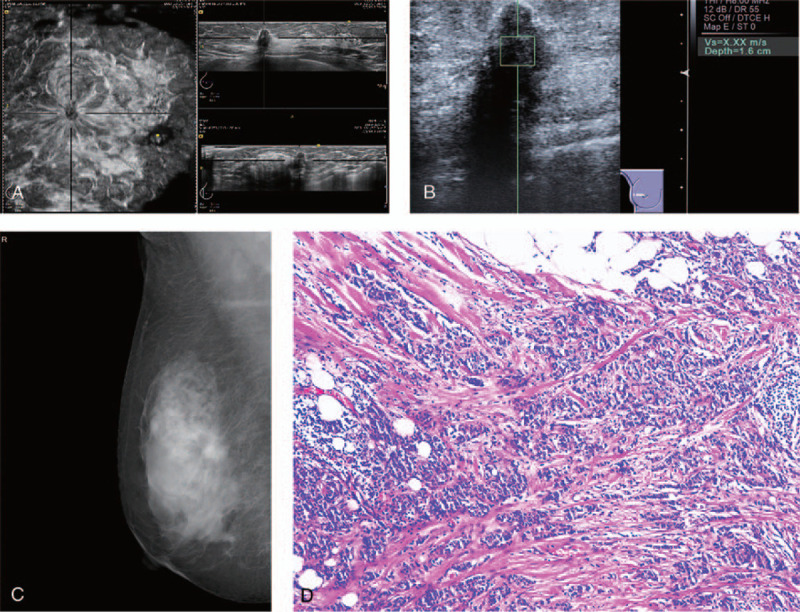
Images of a 47-year-old woman with breast invasive carcinoma of no special type. (A) A solid hypoechoic mass approximately 18 mm × 25 mm in size in the right lateral upper quadrant with a typical “retraction phenomenon” on the coronal surface. (B) Internal value of the shear wave velocity (SWV) was not calculated (displayed as X.XX). (C) Mammogram of the right breast showing an irregular, high-density mass with indistinct margins. (D) The lesion was histopathologically (hematoxylin-eosin staining; original magnification, 200×) confirmed to be invasive carcinoma of no special type.

ABVS is a new breast imaging mode that has been applied in clinical practice in recent years. ABVS can automatically and accurately locate the lesion site and simultaneously obtain coronal images that cannot be displayed by HHUS; ABVS can also transmit image data in the system, providing excellent reproducibility, and a standardized examination mode for clinical practice.^[21]^ The coronal plane is unique to ABVS and unavailable for HHUS. The coronal plane provides additional information for the detection and diagnosis of breast lesions. A recent meta-analysis showed that based on images acquired by ABVS on the transverse, sagittal, and coronal planes simultaneously, ABVS has a high diagnostic accuracy in differentiating benign and malignant breast lesions, with a pooled sensitivity and specificity of 92% (range 89.9–93.8) and 84.9% (range 82.4–87), respectively.^[22]^ The retraction phenomenon is regarded as a strong independent predictor with superior diagnostic performance in differentiating benign and malignant breast lesions (Fig. 3), but its sensitivity is not high.

In the literature, the specificity of the retraction phenomenon may range from 98.4% to 100%, while its sensitivity may be only 39.1% to 70%,^[9,23]^ indicating that most breast lesions with retraction phenomenon signs are malignant, while only partially malignant breast masses exhibit the retraction phenomenon on the coronal plane. In this study, 105 cases of breast masses were pathologically confirmed as nonspecific types of invasive breast cancer, and 68 of these cases in ABVS showed the “retraction phenomenon” on the coronal plane, indicating that the retraction phenomenon with irregular margins on the coronal plane is an important characteristic of invasive breast cancer, which is consistent with previous studies. Lin et al^[24]^ described that the retraction phenomenon had high specificity (100.0%) and high sensitivity (80.0%) in detecting breast cancer and high accuracy (91.4%) in differentiating malignant from benign lesions. Previous studies have reported that the formation of the retraction phenomenon reflects the relationship between the lesion and surrounding tissue. The desmoplastic reaction of breast malignancy can produce contraction of the surrounding tissue toward the mass and disrupt normal parallel tissue planes, which may help explain the generation of this special phenomenon.^[25]^

This study showed that ABVS has a higher detection rate of breast lesions than MG. The ABVS system can display the imaging characteristics and structural features of breast masses in terms of various aspects and on multiple levels, thereby reducing the rate of missed diagnosis. However, MG has a weak ability to penetrate the dense glands and low-fat content of the breast, which can easily cause image overlap, lack of contrast, and omission of some small tumors. In areas with a high incidence of breast cancer in China, dense breasts account for a large proportion, and MG examinations can easily lead to missed diagnosis, especially in the absence of microcalcification.^[26]^ Unaffected by the density of the glands, the comprehensive and multilevel scanning of ABVS helps observe masses at various levels and can identify more concealed lesions, thus compensating for the low detection rate of MG.^[12]^

As an important examination method for the diagnosis of breast lesions, MG is particularly sensitive to calcification and can even detect gritty calcifications with a diameter of 50 μm.^[27]^ Due to the absorption of X-rays, calcifications often appear as bright white spots on X-rays. Because calcification has a good natural contrast with the surrounding tissue, calcification is easy to detect and describe. MG has more advantages in the diagnosis of ductal carcinoma in situ (DCIS) with microcalcification as the main manifestation, which is easily missed by HHUS. The high-resolution images of ABVS can provide a better demonstration of the breast anatomy and proper orientation, enabling the identification of microcalcification in DCIS. In this study, the detection rate of microcalcification using ABVS or MG in breast cancer was similar, and there was no significant difference. The reason may be that dense breasts account for a large proportion of Chinese women, and the low number of DCIS cases in this study led to a decrease in the positive detection rate of MG microcalcification.^[12]^ Additionally, ABVS has a high image resolution and thin layer spacing, that is, only 0.5 mm, and the structure of the whole mammary gland can be observed from multiple perspectives and multi-layer stereoscopics. Furthermore, the post-processing system of the workstation can be employed to adjust the contrast of the image, thereby improving the detection rate of microcalcification by ABVS.^[28]^

ARFI is a shear wave elasticity imaging method that can be used to evaluate tissue elasticity objectively and quantitatively. If the tissue elasticity is good, the SWV value is lower and vice versa. VTQ can be used to quantitatively evaluate the elasticity of the detected region by calculating the shear wave generated by the transverse vibration of the tissue. The more elastic the region, the harder the tissue, and the higher the SWV value.^[29]^ Some studies have shown that ARFI has high value in the differential diagnosis of breast lesions.^[30–32]^ Our study shows that the mean SWV values of 101 benign breast masses and 117 malignant breast masses were 2.69 ± 1.39 meter/second and 7.60 ± 2.61 meter/second, respectively, as measured by VTQ. According to the results of the ROC curve analysis, the difference in the average SWV value between the benign and malignant breast masses was statistically significant when the best diagnostic cutoff value of 3.96 meter/second was used (*t* = 13.893, *P* < .05), which was associated with a sensitivity of 79.06%, specificity of 93.97%, and an AUC of 0.886. Our findings are consistent with those of other studies,^[33–35]^ in which the cutoff value (3.31–4.39 meter/second) was associated with a sensitivity of 67.9% to 88%, specificity of 73% to 93%, and AUC of 0.840 to 0.886. Compared with elastography imaging (EI), ARFI is less operator-dependent, with high diagnostic efficiency and excellent repeatability.^[36]^ This finding indicates that VTQ technology can provide the elastic information of the breast lesion and can be used for the preliminary differential diagnosis of benign and malignant breast masses.

These results suggest that ABVS could be a practical method for detecting breast lesions even if HHUS is not used. With the good reproducibility and high accuracy of ABVS and VTQ, ABVS-VTQ is indicated to have high diagnostic performance in differentiating benign from malignant lesions. Many studies have confirmed that ABVS has good diagnostic performance, with an accuracy of 66% to 97%, specificity of 52.8% to 95%, and sensitivity of 82% to 100%,^[37–41]^ and that ultrasound elastography (UE) has a sensitivity of 78.0% to 100% and specificity of 21.0% to 98.5%.^[41,42]^ Our study shows that the diagnostic performance of ABVS-VTQ (96.33% accuracy, 96.58% sensitivity, and 96.03% specificity) is slightly higher than that of VTQ (86.69% accuracy, 80.34% sensitivity, and 94.05% specificity), ABVS (94.94% accuracy, 94.01% sensitivity, and 96.03% specificity), and MG (69.26% accuracy, 70.08 sensitivity, and 68.31% specificity) alone. ABVS-VTQ has a favorable diagnostic performance.

In this study, 1 case of fat necrosis was mistakenly considered a malignant lesion using each examination method. Fat necrosis is a relatively rare benign disease of the breasts in the clinic. Its imaging features lack specificity and complexity, and thus, it is difficult to distinguish from breast cancer. There are several limitations to our study. The first limitation is the absence of a comparison with HHUS. The case samples collected in this study mainly focus on some common pathological types, such as invasive breast cancer and fibroadenoma, and pathological data of other rare types, especially DCIS, are relatively small. In addition, the ABVS, VTQ, and MG results were analyzed by only 2 radiologists, which may have resulted in operator-related bias. Thus, to explore the application of ABVS-VTQ and MG in some rare pathological types of breast lesions, more case data need to be collected for research and analysis.

## 5 Conclusion

In conclusion, the combined application of ABVS and VTQ in the diagnosis of breast lesions has a higher diagnostic efficiency than MG and can provide more information for the diagnosis of breast lesions. In clinical practice, the combination of ABVS, VTQ, and MG examinations can give full play to the respective advantages of each method simultaneously, which has good application prospects.

## Author contributions

Junli Wang and Banghong Qiang designed the research. Chunyun Shen contributed to the formal analysis and investigation. Yuting Zhu performed the statistical analysis. Hongjie Fan completed the production of the tables and figures. Junli Wang contributed to the paper writing. Yuting Zhu and Banghong Qiang revised the statistical analysis and the manuscript. All authors have read and approved the final manuscript.

**Conceptualization:** Junli Wang, Chunyun Shen.

**Data curation:** Junli Wang, Yuting Zhu.

**Formal analysis:** Chunyun Shen, Banghong Qiang.

**Investigation:** Junli Wang.

**Methodology:** Hongjie Fan, Yuting Zhu, Chunyun Shen.

**Project administration:** Hongjie Fan, Yuting Zhu, Chunyun Shen, Banghong Qiang.

**Software:** Hongjie Fan, Yuting Zhu.

**Supervision:** Hongjie Fan, Banghong Qiang.

**Validation:** Hongjie Fan.

**Visualization:** Hongjie Fan.

**Writing – original draft:** Junli Wang.

**Writing – review & editing:** Hongjie Fan, Banghong Qiang.
